# *N*′-Benzyl­idene­thio­phene-2-carbohydrazide

**DOI:** 10.1107/S1600536810050154

**Published:** 2010-12-08

**Authors:** Jin-He Jiang

**Affiliations:** a Microscale Science Institute, Department of Chemistry and Chemical Engineering, Weifang University, Weifang 261061, People’s Republic of China

## Abstract

In the title compound, C_12_H_10_N_2_OS, the dihedral angle between the phenyl and thio­phene rings is 10.2 (3)°. In the crystal, inversion dimers linked by pairs of N—H⋯O hydrogen bonds generate *R*_2_^2^(8) loops.

## Related literature

For related structures, see: Li & Jian (2010 ▶); Li & Meng (2010 ▶).
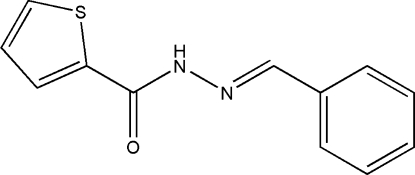


## Experimental

### 

#### Crystal data

C_12_H_10_N_2_OS
*M* *_r_* = 230.28
Monoclinic, 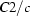

*a* = 22.509 (5) Å
*b* = 5.3202 (11) Å
*c* = 20.855 (4) Å
β = 114.43 (3)°
*V* = 2273.8 (8) Å^3^
*Z* = 8
Mo *K*α radiation
μ = 0.26 mm^−1^
*T* = 293 K
0.22 × 0.20 × 0.18 mm

#### Data collection

Bruker SMART CCD diffractometer
8661 measured reflections
2115 independent reflections
1193 reflections with *I* > 2σ(*I*)
*R*_int_ = 0.073

#### Refinement

*R*[*F* ^2^ > 2σ(*F* ^2^)] = 0.092
*wR*(*F* ^2^) = 0.328
*S* = 1.11
2115 reflections
145 parameters
3 restraints
H-atom parameters constrained
Δρ_max_ = 0.89 e Å^−3^
Δρ_min_ = −0.58 e Å^−3^

### 

Data collection: *SMART* (Bruker, 1997 ▶); cell refinement: *SAINT* (Bruker, 1997 ▶); data reduction: *SAINT*; program(s) used to solve structure: *SHELXS97* (Sheldrick, 2008 ▶); program(s) used to refine structure: *SHELXL97* (Sheldrick, 2008 ▶); molecular graphics: *SHELXTL* (Sheldrick, 2008 ▶); software used to prepare material for publication: *SHELXTL*.

## Supplementary Material

Crystal structure: contains datablocks global, I. DOI: 10.1107/S1600536810050154/hb5756sup1.cif

Structure factors: contains datablocks I. DOI: 10.1107/S1600536810050154/hb5756Isup2.hkl

Additional supplementary materials:  crystallographic information; 3D view; checkCIF report

## Figures and Tables

**Table 1:** Hydrogen-bond geometry (Å, °)

*D*—H⋯*A*	*D*—H	H⋯*A*	*D*⋯*A*	*D*—H⋯*A*
N1—H1*A*⋯O1^i^	0.86	2.04	2.902 (6)	176

Symmetry code: (i) 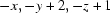.

**Fig. 1.**
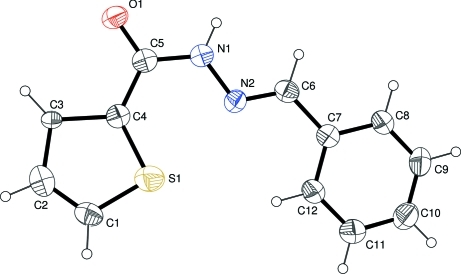
The moleculkar structure of the title compound showing 50% probability displacement ellipsoids.

## supplementary crystallographic information

### Experimental

A mixture of benzaldehyde (0.01 mol) and thiophene-2-carbohydrazide (0.01 mol) was stirred in refluxing ethanol (10 mL) for 2 h to afford the title compound (0.087 mol, yield 87%). Colourless blocks of the title compound were obtained by recrystallization from ethanol at room temperature.

### Refinement

H atoms were fixed geometrically and allowed to ride on their attached atoms, with C—H distances=0.97 Å, and with *U*_iso_=1.2–1.5*U*_eq_.

### Crystal data

**Table:**

C_12_H_10_N_2_OS	*F*(000) = 960
*M**_r_* = 230.28	*D*_x_ = 1.345 Mg m^−^^3^
Monoclinic, *C*2/*c*	Mo *K*α radiation, λ = 0.71073 Å
Hall symbol: -C 2yc	Cell parameters from 2115 reflections
*a* = 22.509 (5) Å	θ = 3.5–25.5°
*b* = 5.3202 (11) Å	µ = 0.26 mm^−^^1^
*c* = 20.855 (4) Å	*T* = 293 K
β = 114.43 (3)°	Block, colorless
*V* = 2273.8 (8) Å^3^	0.22 × 0.20 × 0.18 mm
*Z* = 8	

### Data collection

**Table:**

Bruker SMART CCD diffractometer	1193 reflections with *I* > 2σ(*I*)
Radiation source: fine-focus sealed tube	*R*_int_ = 0.073
graphite	θ_max_ = 25.5°, θ_min_ = 3.5°
phi and ω scans	*h* = −26→26
8661 measured reflections	*k* = −5→6
2115 independent reflections	*l* = −25→25

### Refinement

**Table:**

Refinement on *F*^2^	Primary atom site location: structure-invariant direct methods
Least-squares matrix: full	Secondary atom site location: difference Fourier map
*R*[*F*^2^ > 2σ(*F*^2^)] = 0.092	Hydrogen site location: inferred from neighbouring sites
*wR*(*F*^2^) = 0.328	H-atom parameters constrained
*S* = 1.11	*w* = 1/[σ^2^(*F*_o_^2^) + (0.2*P*)^2^] where *P* = (*F*_o_^2^ + 2*F*_c_^2^)/3
2115 reflections	(Δ/σ)_max_ < 0.001
145 parameters	Δρ_max_ = 0.89 e Å^−^^3^
3 restraints	Δρ_min_ = −0.58 e Å^−^^3^

### Special details

**Table:**

Geometry. All e.s.d.'s (except the e.s.d. in the dihedral angle between two l.s. planes) are estimated using the full covariance matrix. The cell e.s.d.'s are taken into account individually in the estimation of e.s.d.'s in distances, angles and torsion angles; correlations between e.s.d.'s in cell parameters are only used when they are defined by crystal symmetry. An approximate (isotropic) treatment of cell e.s.d.'s is used for estimating e.s.d.'s involving l.s. planes.
Refinement. Refinement of *F*^2^ against ALL reflections. The weighted *R*-factor *wR* and goodness of fit *S* are based on *F*^2^, conventional *R*-factors *R* are based on *F*, with *F* set to zero for negative *F*^2^. The threshold expression of *F*^2^ > σ(*F*^2^) is used only for calculating *R*-factors(gt) *etc*. and is not relevant to the choice of reflections for refinement. *R*-factors based on *F*^2^ are statistically about twice as large as those based on *F*, and *R*- factors based on ALL data will be even larger.

### Fractional atomic coordinates and isotropic or equivalent isotropic displacement parameters (Å^2^)

**Table:**

	*x*	*y*	*z*	*U*_iso_*/*U*_eq_	
S1	0.01403 (9)	0.3874 (4)	0.33888 (9)	0.0815 (8)	
O1	0.06062 (18)	0.7464 (8)	0.51757 (18)	0.0651 (12)	
N2	−0.06627 (19)	0.7700 (9)	0.3491 (2)	0.0503 (11)	
N1	−0.02647 (19)	0.8312 (9)	0.4162 (2)	0.0550 (12)	
H1A	−0.0355	0.9609	0.4352	0.066*	
C7	−0.1623 (2)	0.8729 (10)	0.2456 (3)	0.0506 (13)	
C3	0.1091 (2)	0.3226 (8)	0.4694 (2)	0.0362 (10)	
H3A	0.1359	0.3392	0.5171	0.043*	
C4	0.0476 (2)	0.4805 (10)	0.4224 (3)	0.0501 (13)	
C6	−0.1143 (2)	0.9153 (11)	0.3186 (3)	0.0538 (13)	
H6A	−0.1195	1.0535	0.3431	0.065*	
C5	0.0276 (2)	0.6919 (11)	0.4546 (3)	0.0531 (14)	
C8	−0.2152 (3)	1.0319 (13)	0.2163 (3)	0.0648 (16)	
H8A	−0.2186	1.1702	0.2418	0.078*	
C12	−0.1576 (3)	0.6708 (12)	0.2063 (3)	0.0634 (16)	
H12A	−0.1227	0.5599	0.2254	0.076*	
C10	−0.2577 (3)	0.7903 (14)	0.1120 (3)	0.0783 (19)	
H10A	−0.2905	0.7606	0.0674	0.094*	
C11	−0.2045 (3)	0.6333 (14)	0.1390 (3)	0.0744 (18)	
H11A	−0.2001	0.5019	0.1118	0.089*	
C2	0.1133 (3)	0.1422 (13)	0.4185 (4)	0.0732 (17)	
H2B	0.1459	0.0208	0.4316	0.088*	
C9	−0.2620 (3)	0.9899 (15)	0.1510 (3)	0.081 (2)	
H9A	−0.2976	1.0982	0.1325	0.097*	
C1	0.0680 (3)	0.1595 (12)	0.3516 (3)	0.0683 (17)	
H1B	0.0670	0.0530	0.3158	0.082*	

### Atomic displacement parameters (Å^2^)

**Table:**

	*U*^11^	*U*^22^	*U*^33^	*U*^12^	*U*^13^	*U*^23^
S1	0.0945 (14)	0.0842 (15)	0.0706 (12)	0.0052 (9)	0.0389 (10)	−0.0072 (9)
O1	0.073 (2)	0.066 (3)	0.049 (2)	−0.002 (2)	0.0184 (18)	−0.0075 (18)
N2	0.047 (2)	0.056 (3)	0.047 (2)	−0.001 (2)	0.0179 (18)	0.000 (2)
N1	0.058 (3)	0.056 (3)	0.050 (2)	0.002 (2)	0.020 (2)	−0.003 (2)
C7	0.051 (3)	0.049 (3)	0.055 (3)	−0.003 (2)	0.026 (2)	0.001 (2)
C3	0.040 (2)	0.032 (2)	0.037 (2)	−0.0048 (18)	0.0171 (18)	−0.0054 (18)
C4	0.055 (3)	0.049 (3)	0.050 (3)	−0.005 (2)	0.026 (2)	0.005 (2)
C6	0.053 (3)	0.051 (3)	0.062 (3)	−0.003 (2)	0.029 (2)	−0.005 (3)
C5	0.055 (3)	0.054 (3)	0.055 (3)	−0.009 (2)	0.026 (2)	−0.001 (2)
C8	0.063 (3)	0.058 (4)	0.066 (4)	0.013 (3)	0.019 (3)	−0.004 (3)
C12	0.058 (3)	0.062 (4)	0.066 (3)	0.011 (3)	0.022 (3)	−0.005 (3)
C10	0.077 (4)	0.081 (5)	0.062 (4)	0.000 (4)	0.014 (3)	0.000 (3)
C11	0.076 (4)	0.070 (4)	0.069 (4)	0.003 (3)	0.022 (3)	−0.011 (3)
C2	0.064 (4)	0.067 (4)	0.091 (4)	0.008 (3)	0.034 (3)	0.013 (3)
C9	0.075 (4)	0.086 (5)	0.068 (4)	0.026 (4)	0.017 (3)	0.013 (4)
C1	0.074 (4)	0.069 (4)	0.073 (4)	0.005 (3)	0.042 (3)	−0.011 (3)

### Geometric parameters (Å, °)

**Table:**

S1—C1	1.658 (6)	C6—H6A	0.9300
S1—C4	1.662 (5)	C8—C9	1.352 (8)
O1—C5	1.246 (6)	C8—H8A	0.9300
N2—C6	1.265 (7)	C12—C11	1.378 (8)
N2—N1	1.354 (5)	C12—H12A	0.9300
N1—C5	1.366 (7)	C10—C9	1.366 (10)
N1—H1A	0.8600	C10—C11	1.376 (9)
C7—C8	1.380 (7)	C10—H10A	0.9300
C7—C12	1.382 (8)	C11—H11A	0.9300
C7—C6	1.475 (7)	C2—C1	1.348 (8)
C3—C2	1.463 (8)	C2—H2B	0.9300
C3—C4	1.569 (7)	C9—H9A	0.9300
C3—H3A	0.9300	C1—H1B	0.9300
C4—C5	1.472 (8)		
			
C1—S1—C4	93.7 (3)	C9—C8—H8A	119.5
C6—N2—N1	115.8 (5)	C7—C8—H8A	119.5
N2—N1—C5	121.6 (5)	C11—C12—C7	120.2 (5)
N2—N1—H1A	119.2	C11—C12—H12A	119.9
C5—N1—H1A	119.2	C7—C12—H12A	119.9
C8—C7—C12	118.5 (5)	C9—C10—C11	119.5 (6)
C8—C7—C6	119.6 (5)	C9—C10—H10A	120.2
C12—C7—C6	121.9 (5)	C11—C10—H10A	120.2
C2—C3—C4	101.8 (4)	C10—C11—C12	120.0 (6)
C2—C3—H3A	129.1	C10—C11—H11A	120.0
C4—C3—H3A	129.1	C12—C11—H11A	120.0
C5—C4—C3	118.8 (4)	C1—C2—C3	117.2 (5)
C5—C4—S1	127.9 (4)	C1—C2—H2B	121.4
C3—C4—S1	113.3 (4)	C3—C2—H2B	121.4
N2—C6—C7	122.2 (5)	C8—C9—C10	120.7 (6)
N2—C6—H6A	118.9	C8—C9—H9A	119.7
C7—C6—H6A	118.9	C10—C9—H9A	119.7
O1—C5—N1	119.3 (5)	C2—C1—S1	114.0 (5)
O1—C5—C4	119.8 (5)	C2—C1—H1B	123.0
N1—C5—C4	120.8 (5)	S1—C1—H1B	123.0
C9—C8—C7	121.1 (6)		

### Hydrogen-bond geometry (Å, °)

**Table:**

*D*—H···*A*	*D*—H	H···*A*	*D*···*A*	*D*—H···*A*
N1—H1A···O1^i^	0.86	2.04	2.902 (6)	176

Symmetry codes: (i) −*x*, −*y*+2, −*z*+1.
